# Acoustic analysis of oropharyngeal swallowing using Sonar Doppler^☆^^☆☆^

**DOI:** 10.1016/j.bjorl.2015.12.001

**Published:** 2015-12-17

**Authors:** Franciele Savaris Soria, Roberta Gonçalves da Silva, Ana Maria Furkim

**Affiliations:** a Faculdade Assis Gurgacz, Cascavel, PR, Brazil; b Universidade Estadual de São Paulo (UNESP), São Paulo, SP, Brazil; c Universidade Federal de Santa Catarina (UFSC), Florianópolis, SC, Brazil

**Keywords:** Deglutition, Elderly, Evaluation, Deglutição, Idoso, Avaliação

## Abstract

**Introduction:**

During the aging process, one of the functions that changes is swallowing. These alterations in oropharyngeal swallowing may be diagnosed by methods that allow both the diagnosis and biofeedback monitoring by the patient. One of the methods recently described in the literature for the evaluation of swallowing is the Sonar Doppler.

**Objective:**

To compare the acoustic parameters of oropharyngeal swallowing between different age groups.

**Methods:**

This was a field, quantitative, study. Examination with Sonar Doppler was performed in 75 elderly and 72 non-elderly adult subjects. The following acoustic parameters were established: initial frequency, first peak frequency, second peak frequency; initial intensity, final intensity; and time for the swallowing of saliva, liquid, nectar, honey, and pudding, with 5- and 10-mL free drinks.

**Results:**

Objective, measurable data were obtained; most acoustic parameters studied between adult and elderly groups with respect to consistency and volume were significant.

**Conclusion:**

When comparing elderly with non-elderly adult subjects, there is a modification of the acoustic pattern of swallowing, regarding both consistency and food bolus volume.

## Resumo

**Introdução:**

Durante o processo de envelhecimento, uma das funções que sofre modificação é a deglutição. Estas alterações na deglutição orofaríngea podem ser diagnosticadas por métodos que permitem tanto o diagnóstico quanto o monitoramento e biofeedback ao indivíduo. Um dos métodos descrito na literatura recentemente para a avaliação da deglutição é o Sonar Doppler.

**Objetivo:**

O objetivo desse estudo foi comparar os parâmetros acústicos da deglutição orofaríngea entre faixas etárias distintas.

**Método:**

Estudo de campo, quantitativo. O exame com o Sonar Doppler foi aplicado em 75 idosos e 72 adultos. Estabeleceram-se os parâmetros acústicos: frequência inicial, frequência do primeiro pico, frequência do segundo pico, intensidade inicial, intensidade final e tempo, para as deglutições de saliva, liquido, néctar, mel e pudim, com gole livre, 5 mL e 10 mL.

**Resultados:**

Obtiveram-se dados objetivos e mensuráveis, apresentando significância para a maioria dos parâmetros acústicos estudados entre o grupo de idosos e adultos em relação à consistência e volume.

**Conclusão:**

Verificamos que há modificação do padrão acústico da deglutição, tanto em relação à consistência quanto a volume do bolo alimentar, quando comparados adultos idosos e não idosos.

## Introduction

The world's elderly population is increasing considerably; in 2025, it will exceed the number of children. Therefore, such individuals deserve the attention of interdisciplinary healthcare teams; these professionals need to acquire a better understanding of the aging process and its impact on the individual's health, aiming mainly at improving the quality of life of this population, as well as proposing measures to prevent possible clinical complications.^1^, ^2^

During the aging process, one of the functions that changes is swallowing. Swallowing disorders associated with aging can be conceptualized as presbyphagia.^3^ However, swallowing disturbances caused by neurological and/or structural diseases are called dysphagia; both these disorders and presbyphagia may result in changes of the clinical condition of the patient,^4^, ^5^ possibly with changes in the oral, pharyngeal, and esophageal phases of swallowing. In the oral and pharyngeal phases an increase in the time of bolus transmission occurs, and the same is observed in the esophageal phase, which is associated with a high frequency of non-propulsive contractions.^6^, ^7^

These changes in oropharyngeal swallowing may be diagnosed by methods that allow for both diagnosis and biofeedback monitoring, and thus aid in treatment. These methods include video fluoroscopy, nasal endoscopy, and cervical auscultation.^8^, ^9^

Another method recently described in the literature for the evaluation of swallowing is the Sonar Doppler, which may become a valuable test for assessing swallowing, as it is a painless, noninvasive, and inexpensive test that does not expose the patient to radiation.^10^, ^11^, ^12^

This study aimed to compare the acoustic parameters of oropharyngeal swallowing between different age groups.

## Methods

This research was conducted in two stages. In the first step, a questionnaire (Risk Screening Protocol for Swallowing) that contained questions related to risk factors for dysphagia was administered (Appendix 1). Volunteers who presented risk factors for dysphagia were excluded (with neurological disease, head and neck structural changes, exposition to radiotherapy and/or chemotherapy, and those with swallowing complaints). In total, 189 questionnaires were administered; 147 individuals were selected and participated in the second stage of the research, and were divided into two groups. Group I (GI) consisted of 75 healthy elderly people, aged >60 years, with a mean age of 71 years. Group II (GII) consisted of 72 healthy adult subjects aged between 18 and 59 years, with a mean age of 42 years.

**Figure 1:**
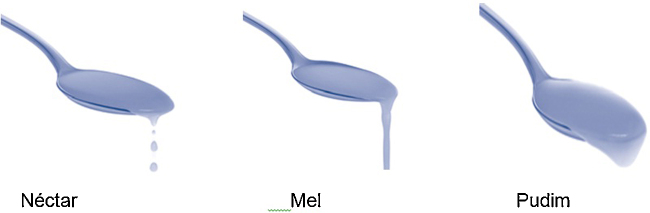
Classification of consistencies – National Dysphagia Diet Guidelines (2002).

In the second phase, the participants were submitted to the evaluation of oropharyngeal swallowing with Sonar Doppler. The assessment followed the protocol proposed by Santos and Macedo-Filho,^10^ with modifications regarding specifications of consistency. The protocol classifies the swallowing of saliva, liquids, and pastes. In the present study, the classification of the National Dysphagia Diet Guidelines (2002)^13^ proposing liquid, nectar, honey, and pudding was used, with the addition of saliva swallows (Fig. 1).

All study subjects received the same food consistency during the procedure, divided into dry swallowing (saliva) and liquid, nectar, honey, and pudding swallowing, using volumes in the sequence described: 5 mL-, 10 mL-, and free swallows. In the sequence described, four swallows were required: firstly, saliva swallowing, followed by free-, 5 mL- and 10 mL-swallows of each consistency.

The consistencies were prepared with the Nutillis^®^ thickener (a food thickener consisting of corn starch and food gums, manufactured by Support^®^) and offered immediately after preparation, according to the recommendations of the National Dysphagia Diet Guidelines.^13^

**Figure 2:**
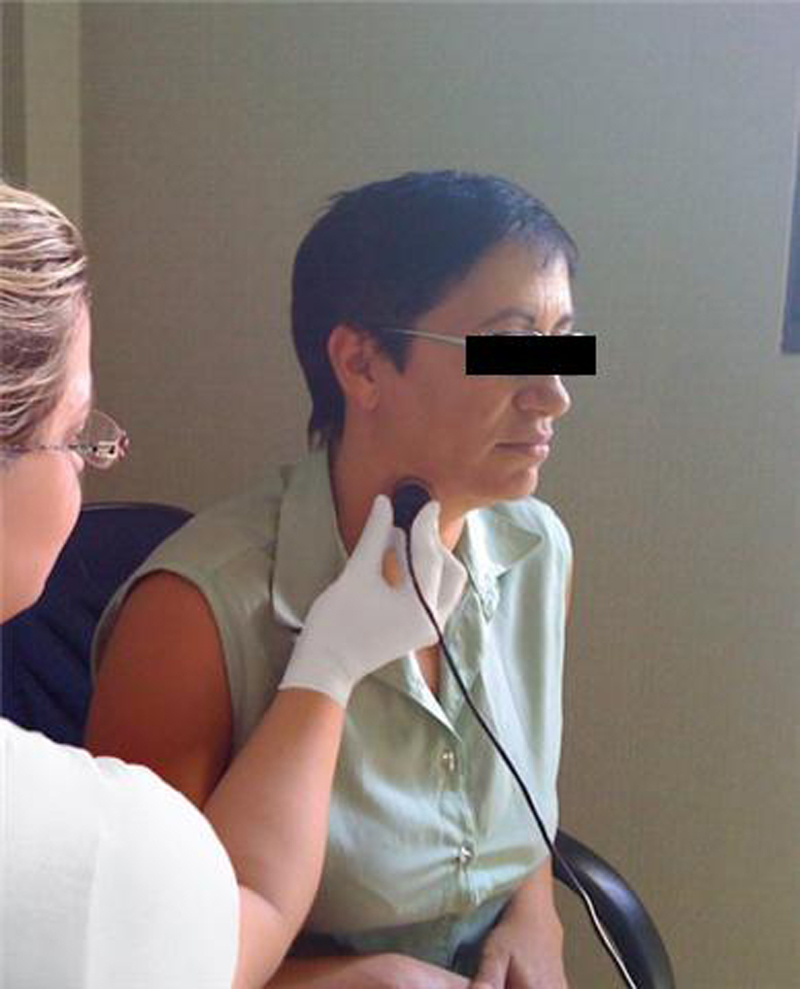
Transducer placement position in the patient.

In the process of capturing swallowing sounds by Sonar Doppler, the subject tested remained in a seated position and with a free neck. The transducer was placed in the lateral region of the trachea immediately below the cricoid on the right side, and the transducer beam was positioned to form an angle of 30–60^14^ (Fig. 2).

**Figure 3:**
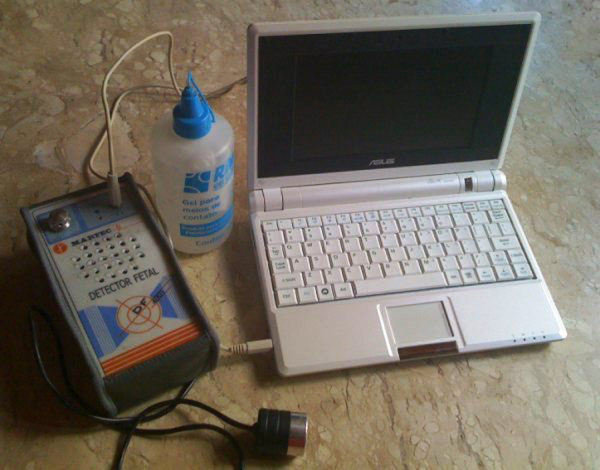
Ultrasonic detector (portable), DF-4001, Martec.

**Figure 4:**
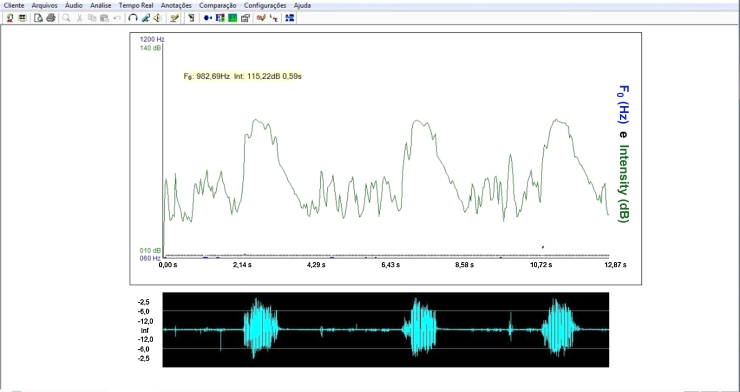
Flat disk transducer.

The equipment used was a portable ultrasonic detector (DF-4001 model; Martec; Fig. 3). A single-crystal, flat disk transducer provided the Doppler interface (Fig. 4). Contact gel was used on the transducer to facilitate skin contact (Fig. 3). Ultrasound frequency (by Doppler effect) was set at 2.5 MHz; output, 10 mW/cm^2^; sound output power, 1 W. The equipment was connected to a computer (Fig. 3).

For the acoustic analysis of the sound signal captured by the sonar, the VoxMetria software (Fig. 4) was used. To capture sound signals by the continuous Doppler equipment, the machine's volume was adjusted to No. 3. The intensity values were analyzed with a lower limit = 10 dB and an upper limit = 140 dB.

**Figure 5:**
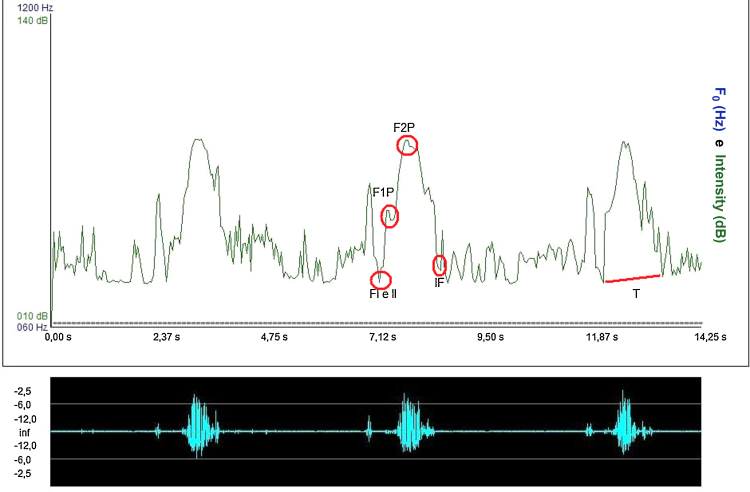
Interface of Voxmetria software.

The acoustic parameters studied followed the same parameters proposed by Santos and Macedo-Filho,^10^ namely:

Initial frequency (IF) of sound signal: defined as the first tracing of the sound wave, representing the onset of swallowing^15^;
Frequency of the first peak (F1P): defined as the first peak observed on the sound wave of swallowing, representing laryngeal elevation^15^;
Frequency of the second peak (F2P): defined as the second peak of the sound wave of swallowing, representing cricopharyngeal opening^15^;
Initial intensity (II): defined as the initial intensity of the acoustic plotted line recorded by Doppler during the beginning of the swallowing event.^15^ The intensity values were analyzed with a lower limit of 10 dB and an upper limit of 140 dB.
Final intensity (FI): defined as the end of the second wave peak recorded by Doppler during the swallowing event, obtaining the amplitude of the audio signal. It is the weak signal, associated with the descent of the larynx after swallowing.^15^ The intensity values were analyzed with a lower limit of 10 dB and an upper limit of 140 dB.
Acoustic time (T): defined as the time interval between the point of apnea of deglutition (FI)^16^ to post-swallowing glottal expiratory release (Fig. 5).

The statistical methods used in the study were the inferential technique and significance test. To analyze the significance of data obtained from acoustic parameters between elderly and adult groups in each consistency and in each volume, Student's t-test – equal variance of two samples was used, and the significance level was set at 0.05. In the statistical analysis, a crossover between elderly (GI) and adult (GII) groups was carried out, comparing the parameters proposed in the method.

This study was approved by the Research Ethics Committee, under No. 00061/2008.

## Results

There was no statistically significant difference between groups in the analysis of the initial and final frequencies, either for the different consistencies used or the different volumes (Table 1, Table 2). A significant difference between the groups analyzed was detected in all parameters of the first and second peaks for the different consistencies, with few exceptions (Table 3, Table 4).

**Table 1:** Comparison between elderly (GI) and adult (GII) groups for initial frequency (IF).

Consistency	Elderly group	Adult group	*p*-value
Dry	350.6 Hz	578.2 Hz	0.0000^a^
Liquid – DL	404.8 Hz	567.4 Hz	0.0000^a^
Liquid – 5 mL	556.8 Hz	571.5 Hz	0.19610
Liquid – 10 mL	473.1 Hz	684.4 Hz	0.0000^a^
Nectar – DL	406.4 Hz	616.7 Hz	0.0000^a^
Nectar – 5 mL	566.1 Hz	404.5	0.0000^a^
Nectar – 10 mL	489.7 Hz	471.9 Hz	0.1034
Honey – DL	416.4 Hz	603.0 Hz	0.0000^a^
Honey – 5 mL	560.1 Hz	587.9 Hz	0.0093^a^
Honey – 10 mL	429.1 Hz	384.1 Hz	0.0020^a^
Pudding – DL	412.8 Hz	333.0 Hz	0.0000^a^
Pudding – 5 mL	569.7 Hz	555.3 Hz	0.1097
Pudding – 10 mL	433.7 Hz	342.3 Hz	0.0000^a^

Student's *t*-test.

a Significant differences at the 0.05 significance level.

**Table 2:** Comparison between the elderly (GI) and adult (GII) groups for frequency of first peak (F1P).

Consistency	Elderly group	Adult group	*p*-value
Dry	661.9 Hz	509.1 Hz	0.0001^a^
Liquid – DL	831.4 Hz	916 Hz	0.0000^a^
Liquid – 5 mL	832.3 Hz	887.8 Hz	0.0000^a^
Liquid – 10 mL	793.6 Hz	1010.9 Hz	0.0000^a^
Nectar – DL	779.8 Hz	833.8 Hz	0.0000^a^
Nectar – 5 mL	810.7 Hz	799.0 Hz	0.2416
Nectar – 10 mL	990.1 Hz	1050.2 Hz	0.0001^a^
Honey – DL	800.6 Hz	897.1 Hz	0.0000^a^
Honey – 5 mL	813.7 Hz	819.5 Hz	0.4761
Honey – 10 mL	890.0 Hz	354.5 Hz	0.0000^a^
Pudding – DL	791.8 Hz	802.9 Hz	0.4634
Pudding – 5 mL	828.2 Hz	743.5 Hz	0.0000^a^
Pudding – 10 mL	886.0 Hz	891.2 Hz	0.6159

Student's *t*-test.

a Significant differences at the 0.05 significance level.

**Table 3:** Comparison between the elderly (GI) and adult (GII) groups for frequency of second peak (F2P).

Consistency	Elderly group	Adult group	*p*-value
Dry	870.1 Hz	1005.5 Hz	0.0000^a^
Liquid – DL	1054.9 Hz	1043.9 Hz	0.0697
Liquid – 5 mL	967.8 Hz	1041.7 Hz	0.0000^a^
Liquid – 10 mL	977.9 Hz	1078.4 Hz	0.0000^a^
Nectar – DL	1042.3 Hz	967.2 Hz	0.0000^a^
Nectar – 5 mL	980.9 Hz	978.6 Hz	0.7994
Nectar – 10 mL	1155.4 Hz	1102.8 Hz	0.0000^a^
Honey – DL	1045.6 Hz	1062.0 Hz	0.0050^a^
Honey – 5 mL	974.6 Hz	966.7 Hz	0.2646
Honey – 10 mL	1087.5 Hz	1045.0 Hz	0.0000^a^
Pudding – DL	1046.0 Hz	1032 Hz	0.0191^a^
Pudding– 5 mL	976.3 Hz	950.7 Hz	0.0000^a^
Pudding – 10 mL	1029.4 Hz	1038.4 Hz	0.0260^a^

Student's *t*-test.

a Significant differences at the 0.05 significance level.

**Table 4:** Comparison between elderly (GI) and adult (GII) groups for initial intensity (II).

Consistency	Elderly group	Adult group	*p*-value
Dry	34.8 dB	52.4 dB	0.0000^a^
Liquid – DL	38.6 dB	5.3 dB	0.0000^a^
Liquid – 5 mL	43.5 dB	45.5 dB	0.0611
Liquid – 10 mL	48.9 dB	65.3 dB	0.0000^a^
Nectar – DL	38.0 dB	29.3 dB	0.0000^a^
Nectar – 5 mL	44.0 dB	32.6 dB	0.0000^a^
Nectar – 10 mL	36.8 dB	32.4 dB	0.0000^a^
Honey – DL	38.1 dB	54.2 dB	0.0000^a^
Honey – 5 mL	44.5 dB	40.3 dB	0.0002^a^
Honey – 10 mL	41.4 dB	44.1 dB	0.0227^a^
Pudding – DL	38.2 dB	38.2 dB	0.8278
Pudding – 5 mL	44.9 dB	42.5 dB	0.1530
Pudding – 10 mL	36.7 dB	36.2 dB	0.7228

Student's *t*-test.

a Significant differences at the 0.05 significance level.

In most of the comparisons carried out on the initial and final intensities, statistical significance among groups and consistencies was observed (Table 4, Table 5). All comparisons were statistically significant for the time parameter (Table 6).

**Table 5:** Comparison between the elderly (GI) and adult (GII) groups for final intensity (FI).

Consistency	Elderly group	Adult group	*p*-value
Dry	73.2 dB	4.7 dB	0.0000^a^
Liquid – DL	87.7 dB	2.5 dB	0.0000^a^
Liquid – 5 mL	84.1 dB	86.6 dB	0.0068^a^
Liquid – 10 mL	36.2 dB	73.0 dB	0.0000^a^
Nectar – DL	87.7 dB	73.2 dB	0.0000^a^
Nectar – 5 mL	84.0 dB	76.8 dB	0.0000^a^
Nectar – 10 mL	43.6 dB	39.1 dB	0.0000^a^
Honey – DL	87.9 dB	88.9 dB	0.0340^a^
Honey – 5 mL	84.1 dB	75.0 dB	0.0000^a^
Honey – 10 mL	40.4 dB	43.9 dB	0.0012^a^
Pudding – DL	87.7 dB	76.6 dB	0.0000^a^
Pudding – 5 mL	83.9 dB	75.6 dB	0.0000^a^
Pudding – 10 mL	31.6 dB	30.4 dB	0.1312

Student's *t*-test.

a Significant differences at the 0.05 significance level.

**Table 6:** Comparison between the elderly (GI) and adult (GII) groups for time (T).

Consistency	Elderly group	Adult group	*p*-value
Dry	1.7 s	0.2 s	0.0000^a^
Liquid – DL	1.5 s	0.3 s	0.0000^a^
Liquid – 5 mL	1.4 s	0.2 s	0.0000^a^
Liquid – 10 mL	1.7 s	1.6 s	0.0000^a^
Nectar – DL	1.5 s	1.2 s	0.0000^a^
Nectar – 5 mL	1.4 s	1.3 s	0.0256^a^
Nectar – 10 mL	1.7 s	1.5 s	0.0040^a^
Honey – DL	1.5 s	1.8 s	0.0000^a^
Honey – 5 mL	1.4 s	1.3 s	0.0256^a^
Honey – 10 mL	1.7 s	1.4 s	0.0256^a^
Pudding – DL	1.7 s	1.5 s	0.0040^a^
Pudding – 5 mL	1.4 s	1.3 s	0.0256^a^
Pudding – 10 mL	1.6 s	1.4 s	0.0000^a^

Student's *t*-test.

a Significant differences at the 0.05 significance level.

## Discussion

Because it is relatively low cost, the Doppler Sonar compares favorably with other tests, such as nasolaryngofibroscopy and video fluoroscopy. Moreover, it is not invasive, does not require sedation, is painless, and does not expose patients to radiation.

In the present study, specific characteristics of the sound curves evaluated with Sonar Doppler showed that there are significant differences in the swallowing patterns of healthy elderly subjects compared to healthy non-elderly adults.

But it was not possible to compare the present data with that from other studies, because of the originality of this research. However, this study opens a reference database for future research, and provides normal acoustic parameters for sound waves during swallowing in two different age groups.

In the elderly population, some changes were observed during swallowing. The elderly often have reduced functional reserves of various organs and systems, and this includes changes in the phases of deglutition. When these individuals are free of health problems, they make use of compensatory strategies, such as the use of strength during swallowing and increased tongue pressure into the oral cavity, attempting to assist the propulsion of food.^17^, ^18^, ^19^

In agreement with the literature, this study identified a higher incidence of decreased strength, increased time, and a slower adaptation to different consistencies in the deglutition of the elderly, compared to non-elderly adult subjects.^20^

The initial frequency (IF) and the initial intensity (II), which represent the beginning of swallowing,^15^ were less intense in elderly than in adult subjects – that is, the former group presented less muscle strength and/or a decrease in speed at the onset of pharyngeal phase.

Conversely, the frequency of the first peak (F1P), which characterizes laryngeal elevation,^15^ was of greater intensity in the elderly than in the adult subjects. One hypothesis for this may be related to a decrease of salivation in the elderly, with a lower volume of saliva. Therefore, elderly subjects need more strength to accomplish laryngeal elevation.

The intensity for the second peak (F2P), which represents cricopharyngeal opening,^15^ was decreased in the elderly, possibly due to a muscular slowing observed in this population for carrying out this opening.^21^, ^22^ The results on the final intensity (FI), which characterizes the laryngeal descent at the end of swallowing,^15^ may have occurred due to a reduced laryngeal elevation found in senescent subjects, and thus the laryngeal descent would be smaller, requiring less strength.^23^

As described by several authors, the swallowing time was longer in the elderly as a consequence of a slower swallowing process, due to the characteristics of presbyphagia.^22^, ^24^

No statistically significant difference was noted between the groups during the process of swallowing liquids (with drink), but the frequency of the first peak (F1P) was less intense in the elderly. This finding may be explained by a decrease in the degree of laryngeal elevation, pertinent to the aging process – a finding that parallels those described in the literature.^25^

All differences found in this study between deglutition of the elderly and healthy adults are in agreement with the literature about this population, which reports a slowing of muscle movements, cricopharyngeal sphincter and pharyngeal closure dysfunction, reduced laryngeal elevation, and an increase in swallowing time during the deglutition process of healthy elderly people (thus with presbyphagia).^26^, ^27^

In the aging process, there are differences in relation to the events, and how these differences impact individuals. The development of swallowing takes place heterogeneously, and an ability to adapt is the main feature of a healthy aging process; this may be one possible explanation for the lack of significance in the results between the elderly and the non-elderly adults.^20^, ^28^

The multiple characteristics of swallowing sounds depend directly on the food consistency: and an increase in food consistency causes difficulty in the preparation and organization of the food bolus, its slow handling, ejection difficulties, and a decrease in the anteroposterior movement of the tongue. Therefore, both the consistency and volume of food interfere in the swallowing process.^11^, ^29^, ^30^

The main feature observed in the elderly was a curve with smaller amplitude and longer duration compared with adult subjects. These data suggest that, in the elderly besides being slower, the swallowing process follows a broader morphofunctional accommodation in terms of mobility; however, the swallowing process is effective and competent in this population.

It is critical to carry out further studies using this methodology, but with the addition of examinations using imaging technology, in order to standardize the curves and simultaneously analyze the sound and image of the swallowing process with specific software.

In most studies on acoustic analysis of deglutition, the relationship between acoustic findings and physiological events of the swallowing process cannot be clarified. The structural and functional correlation of these two events may enable a more accurate diagnosis, aiding in more specific therapeutic approaches and also facilitating the standardization of these acoustic parameters of swallowing.

## Conclusion

There is a modification of the acoustic pattern of swallowing, both with regard to consistency and to bolus volume in elderly subjects compared to non-elderly adult subjects.

## Conflicts of interest

The authors declare no conflicts of interest.

## Figures and Tables

**Table:** 

**Questionnaire 1**	
*1. Name:*	

*2. Gender:*	
() Female	() Male

*3. Age:*	

*4. Previous diseases:*	

*5. Have you ever been exposed to chemotherapy and/or radiotherapy?*	
() YES	() NO

*6. Have you ever been treated for head and/or neck conditions?*	
() YES	() NO

*7. Do you have any structural changes of head and/or neck?*	
() YES	() NO

*8. Do you feel difficulty to swallow?*	
() YES	() NO

*9. Do you feel fatigue during meals?*	
() YES	() NO

*10. Do you cough during or after meals?*	
() YES	() NO

*11. Do you experience a feeling of “wet voice” after meals?*	
() YES	() NO

*12. Do you experience a feeling of food stopping in the throat?*	
() YES	() NO

*13. Do you experience pain or discomfort when swallowing food?*	
() YES	() NO
